# *Brassica rapa* orphan gene *BR1* delays flowering time in *Arabidopsis*

**DOI:** 10.3389/fpls.2023.1135684

**Published:** 2023-02-24

**Authors:** Mingliang Jiang, Yuting Zhang, Xiaolong Yang, Xiaonan Li, Hong Lang

**Affiliations:** ^1^ School of Agriculture, Jilin Agricultural Science and Technology College, Jilin, China; ^2^ College of Horticulture, Shenyang Agricultural University, Shenyang, China; ^3^ College of Horticulture, South China Agricultural University, Guangzhou, China

**Keywords:** *Brassica rapa*, orphan gene, *BR1*, bolting resistance, *Arabidopsis*

## Abstract

Orphan genes are essential to the emergence of species-specific traits and the process of evolution, lacking sequence similarity to any other identified genes. As they lack recognizable domains or functional motifs, however, efforts to characterize these orphan genes are often difficult. Flowering is a key trait in *Brassica rapa*, as premature bolting can have a pronounced adverse impact on plant quality and yield. Bolting resistance-related orphan genes, however, have yet to be characterized. In this study, an orphan gene designated *BOLTING RESISTANCE 1* (*BR1*) was identified and found through gene structural variation analyses to be more highly conserved in Chinese cabbage than in other available accessions. The expression of *BR1* was increased in bolting resistant Chinese cabbage and decreased in bolting non-resistant type, and the expression of some mark genes were consist with bolting resistance phenotype. *BR1* is primarily expressed in leaves at the vegetative growth stage, and the highest *BR1* expression levels during the flowering stage were observed in the flower buds and silique as compared to other tissue types. The overexpression of *BR1* in *Arabidopsis* was associated with enhanced bolting resistance under long day (LD) conditions, with these transgenic plants exhibiting significant decreases in stem height, rosette radius, and chlorophyll content. Transcriptomic sequencing of WT and *BR1*OE plants showed the association of *BR1* with other bolting resistance genes. Transcriptomic sequencing and qPCR revealed that six flowering integrator genes and one chlorophyll biosynthesis-related gene were downregulated following *BR1* overexpression. Six key genes in photoperiodic flowering pathway exhibited downward expression trends in *BR1*OE plants, while the expression of floral repressor *AtFLC* gene was upregulated. The transcripts of these key genes were consistent with observed phenotypes in *BR1*OE plants, and the results indicated that *BR1* may function through vernalization and photoperiodic pathway. Instead, the protein encoded by *BR1* gene was subsequently found to localize to the nucleus. Taken together, we first propose that orphan gene *BR1* functions as a novel regulator of flowering time, and these results suggested that *BR1* may represent a promising candidate gene to support the selective breeding of Chinese cabbage cultivars with enhanced bolting resistance.

## 1 Introduction

Orphan genes (*OGs*) are species- or lineage-specific genes that lack sequence similarity with other known genes expressed by other species (Jiang et al., 2022), often arising as a result of rapid evolutionary activity (Cai et al., 2006). To date, *OGs* have been reported in many genomic sequencing analyses of species including *Aegiceras corniculatum* (Ma et al., 2021), *Arabidopsis thaliana* (Donoghue et al., 2011), *Brassica rapa* (Jiang et al., 2018), eight Cucurbitaceae species (Ma et al., 2022), *Oryza sativa* (Cui et al., 2015), and *Vigna unguiculata* (Li et al., 2019). As *OGs* do not contain recognizable domains, functional motifs, or folding patterns, their functions are often unclear such that detailed functional characterization is ultimately necessary. Several studies have successfully demonstrated the diverse roles played by specific *OGs* as mediators of metabolite synthesis (Li et al., 2009; Li and Wurtele, 2015; Li et al., 2015b; O’Conner et al., 2018; Jiang et al., 2020b; Fang et al., 2021; Shen et al., 2021; Tanvir et al., 2022b), biotic stresses response (Jiang et al., 2018; Qi et al., 2019; Brennan et al., 2020; Jiang et al., 2020a; Wang et al., 2021; Moon et al., 2022; Tanvir et al., 2022a), abiotic stresses response (Yadeta et al., 2014; Li et al., 2019; Ma et al., 2020), species-specific traits, or the regulation of growth and development (Chen et al., 2017; Ni et al., 2017; Wang et al., 2017; Zhao et al., 2018; Dossa et al., 2021). These characteristics make *OGs* important targets for plant breeding efforts aimed at enhancing specific traits of interest to improve plant stress resistance and quality. However, the ability of specific *OGs* to regulate the timing of flowering has largely been overlooked to date.

Flowering timing is an agronomically important trait that can determine reproductive success and shape crop yields. This timing is thus regulated by a complex network of signaling proteins and processes that are responsive to external stimuli and developmental cues (Wang, 2014; Blümel et al., 2015). Five genetically defined pathways have been identified to date as important regulators of floral transition, including the age, photoperiod, hormone, autonomous, and vernalization pathways (Srikanth and Schmid, 2011; Li et al., 2015a; Freytes et al., 2021). These pathways integrate diverse signaling inputs associated with flowering and ultimately regulate the expression of important genes that govern flowering timing including *APETALA1* (*AP1*, *AT1G69120*), *SUPPRESSOR OF OVEREXPRESSION OF CO1* (*SOC1*, *AT2G45660*), *FLOWERING LOCUS T* (*FT*, *AT1G65480*), and the plant-specific transcription factor (TF) *LEAFY* (*LFY*, *AT5G61850*) (Wang, 2014; Bao et al., 2020). The autonomous pathway can promote flowering in a manner that is independent of the length of the day through the suppression of *FLOWERING LOCUS C* (*FLC*, *AT5G10140*), which is a central repressor of flowering and vernalization (Simpson and Dean, 2002; Jung and Müller, 2009; Cheng et al., 2017). FLC is an important inhibitor of flowering activity that can suppress shoot apical meristem (SAM) floral transition-related TF expression (Cho et al., 2017). In the photoperiod pathway, daily patterns of expression for the floral regulators *CONSTANS* (*CO*, *AT5G15840*) and *FT* are regulated by a range of positive and negative factors that can influence protein-protein interactions, chromatin structural characteristics, protein stability, and transcriptional activity (Johansson and Staiger, 2015). Circadian rhythms are closely linked to the photoperiod-mediated control of the transition from vegetative to reproductive plant growth (Creux and Harmer, 2019). Gibberellin (GA) pathway signaling serves as a major hormonal mechanism that regulates flowering activity, although other hormones including jasmonate, brassinosteroid, abscisic acid, cytokinins, and ethylene also play regulatory roles in this context (Izawa, 2021). The *miR156*-*SPL* (*SQUAMOSA PROMOTER BINDING PROTEIN-LIKE*) and *miR172*-*AP2* (*APETALA2*, *AT4G36920*) modules have been suggested as key regulatory hubs involved in the age pathway that facilitate plant flowering under non-inductive conditions (Wang, 2014; Kinoshita and Richter, 2020). While these results provide important insight into the mechanisms that control the timing of flowering, the mechanistic links among these pathways and the regulatory crosstalk between them have yet to be characterized in detail.

Heading is among the most important agronomic traits for *B. rapa* ssp. *pekinensis* (Chinese cabbage) or *Brassica oleracea* var. *capitata* (cabbage) and has been the subject of extensive research interest (Zhang et al., 2022). Premature bolting can have a severe adverse impact on Chinese cabbage or cabbage yields and quality, restricting the geographic distribution and planting season for this economically important species (Su et al., 2018). Accordingly, there is a pressing need to breed novel cultivars with enhanced bolting resistance. The histone H4 protein encoded by *BrHIS4.A04* (*Bra035673*) has previously been shown to attenuate photoperiod-related flowering gene expression under drought conditions in Chinese cabbage plants *via* signaling through the ABA pathway, thus preventing premature bolting (Xin et al., 2020). *SET DOMAIN GROUP 8* (*BrSDG8*, *BraA07g040740.3C*) serves as an additional regulator of early bolting in *B. rapa* ssp. *pekinensis*, with *FLC* H3K6 methylation activity increasing when the function of this gene is disrupted (Fu et al., 2020). *BrFLC5* (*Bra022771*) was also previously found to be expressed at lower levels than two other *BrFLC* genes, supporting efforts to breed *B. rapa* plants resistant to premature bolting (Xi et al., 2018). Cabbage *BoFLC4-1* played a similar role to *Arabidopsis FLC* in regulating flowering time (Lin et al., 2005). The intron I 215-bp indel of *BoFLC2* influenced the flowering time of cabbage, which might offer critical information to promote the study of epigenetic gene silencing processes in flowering-related genes (Li et al., 2022). Study also showed that *BoFLC1*, *BoFLC3*, and *BoFLC5* were within the confidence intervals of known flowering time quantitative trait loci (QTL) (Razi et al., 2008). These prior results thus offer valuable insights that can be leveraged to better facilitate the genetic control of the bolting and flowering processes.

In this study, the *OG* designated *BOLTING RESISTANCE 1* (*BR1*) was the target of functional characterization efforts exploring its relationship with the timing of flowering. Structural genotypic variations and sequence characteristics in the *BR1* gene region were analyzed in 524 *B. rapa* accessions, and its expressions in bolting resistant or bolting non-resistant Chinese cabbage were detected. Representative mark genes were determined in bolting resistant type inbred lines. And *BR1* expression patterns were examined over the course of Chinese cabbage development in different tissue compartments. The impact of *BR1* overexpression on flowering time was additionally assessed in *Arabidopsis thaliana*, while transcriptomic sequencing was used to explore the mechanistic basis for the ability of *BR1* to delay *A. thaliana* flowering time. Subcellular localization of the BR1 protein was additionally analyzed. Together, these analyses identified the *B. rapa* ssp. *pekinensis BR1* gene as a key regulator of delayed flowering time in *A. thaliana*.

## 2 Materials and methods

### 2.1 Plant material and cultivation

*B. rapa* ‘Chiifu’ cultivar, Chinese cabbage inbred lines, *A. thaliana* ecotype ‘Columbia-0’ (Col-0), and the transgenic *A. thaliana* lines were cultivated as in our prior studies (Jiang et al., 2018; Jiang et al., 2020b). *Nicotiana benthamiana* was cultivated as in prior study (Zhan et al., 2022).

### 2.2 *BR1* sequence analyses

The CD-Search tool was used to search the NCBI Conserved Domain Database (https://www.ncbi.nlm.nih.gov/Structure/cdd/wrpsb.cgi) for conserved domains in BR1. The SignalP 5.0 server (https://services.healthtech.dtu.dk/service.php?SignalP-5.0) was used to identify predicted signal peptide sequences in BR1. The PROSITE database (https://prosite.expasy.org/) was used to predict motifs in BR1, while TF predictions were made with the Plant Transcription Factor Database (PlantTFDB v5.0, http://planttfdb.gao-lab.org/). Genotypic analyses of structural variations in the *BR1* gene region were performed by comparing 524 *B. rapa* accessions with the Polymorph tool using the Brassicaceae Database (BRAD, http://brassicadb.cn/) as reported previously (Cai et al., 2021), with these 524 different *B. rapa* accessions being derived from a separate report (Cheng et al., 2016; Su et al., 2018; Cai et al., 2021). Ten orphan genes analyzed in this study were identified in our previous study (Jiang et al., 2018). The *B. rapa* genome version 3.0 was used for structural variation analyses.

### 2.3 Analyses of *BR1* expression profiles in Chinese cabbage

Quantitative real-time PCR (qPCR) analyses were performed as detailed previously (Jiang et al., 2018), using primers compiled in **Supplementary Table S1**. Sampling of plants at the seedling and flowering stages was performed as published previously (Jiang et al., 2018), while sampling at the rosette and heading stages was performed at the 6^th^ and 8^th^ weeks, respectively. When rosette stage sampling was performed, three leaves were collected from each of nine individual ‘Chiifu’ plants (three biological replicates, three plants per replicate). With the oldest leaf numbered as leaf one, samples were collected from different positions (top, middle, bottom) on three different leaves (outer leaf, first leaf; middle leaf, 10^th^ leaf; inner leaf, 20^th^ leaf), designated as the RL1, RL2, and RL3 from the outer to the inner leaves. In the heading stage, three leaves (outer leaf, first leaf; middle leaf, 20^th^ leaf; inner leaf, 40^th^ leaf) were similarly collected from each of nine ‘Chiifu’ plants, with these samples being respectively designated as HL1, HL2, and HL3. For *BR1* expression in bolting resistant or bolting non-resistant Chinese cabbage, ten inbred lines from heading stage were selected from our laboratory, the top point of short stem (GP) and the top point of inner leaf (TP) were sampled, TP was used as control. Three biological replicates with three plants of different lines per replicate were sampled. After collection, samples were snap-frozen with liquid nitrogen and stored at −80°C for subsequent RNA isolation. qPCR primers for the expression analyses of representative mark genes were listed in **Supplementary Table S1**.

### 2.4 Establishment and analysis of *BR1*-overexpressing transgenic *Arabidopsis* plants

Vector constructs, *Arabidopsis* transformation, and selection were all performed as detailed in our prior report (Jiang et al., 2020b). Vector construction was performed using primer pairs compiled in **Supplementary Table S1**. Phenotypic analyses of transgenic *Arabidopsis* plants were performed as detailed in our prior study (Jiang et al., 2020b).

### 2.5 Transcriptomic sequencing and validation

Three biological replicate samples were collected from the aerial portions of WT and *BR1*OE mutant plants 25 days post-planting. After snap freezing using liquid nitrogen, these samples were stored at −80°C. RNA extraction was performed as detailed previously (Jiang et al., 2018), and 1% agarose gel electrophoresis was used to detect any RNA contamination or degradation while a NanoPhotometer^®^ instrument (IMPLEN, CA, USA) was used to confirm RNA purity. A Qubit^®^ RNA Assay Kit and a Qubit^®^ 2.0 Fluorometer (Life Technologies, CA, USA) were used to quantify the RNA concentrations in individual samples, while an RNA Nano 6000 Assay Kit and a Bioanalyzer 2100 instrument (Agilent Technologies, CA, USA) were used to confirm RNA integrity. Sequencing libraries were prepared from 1 μg of RNA per sample with a NEBNext^®^ UltraTM RNA Library Prep Kit for Illumina^®^ (NEB, USA) based on provided directions. Library sequencing was then performed with an Illumina Hiseq platform to generate 125 bp/150 bp paired-end reads.

Initial data were filtered with Fastp (v0.19.3) to remove adapter-containing reads, reads containing > 10% N bases, and reads with > 50% low-quality (Q ≤ 20) bases. The clean reads were then compared to the *Arabidopsis* TAIR10 genome which was downloaded from The *Arabidopsis* Information Resource (TAIR) (https://www.arabidopsis.org/) using HISAT (v2.1.0). New gene predictions were made using StringTie (v1.3.4d), while gene alignment was calculated with FeatureCounts (v1.6.2), and fragments per kilobases of exons per million mapped reads (FPKM) expression values were then calculated for all transcripts. Differentially expressed genes (DEGs) were identified using DESeq2 (v1.22.1) based on Benjamini & Hochberg-corrected *p*-values, a |log2Fold Change| ≥ 1, and a false discovery rate (FDR) < 0.05. Hypergeometric tests were used for Gene Ontology (GO) term and KEGG pathway enrichment analyses. Gene expression was validated using primer pairs listed in **Supplementary Table S1**.

### 2.6 Subcellular localization analyses

The *BR1* coding sequence was cloned into the pCAM35-GFP vector without the corresponding stop codons using the KpnI and BamHI cleavage sites. pCAM35::BR1::GFP expression vector construction and *N. benthamiana* epidermal cell infection was performed as reported previously (Zhang et al., 2021). Nuclei were visualized through the co-expression of a mCherry-labeled nuclear marker. All experiments were independently repeated in triplicate, and a Leica confocal microscope (SP8, Germany) was used to visualize cells at 48 h following agro-infiltration. Primer pairs used in vector construction are compiled in **Supplementary Table S1**.

### 2.7 Statistical analysis

SPSS 19.0 was used to compare data through Student’s *t*-tests or one-way ANOVAs with Duncan’s multiple range test as appropriate.

## 3 Results

### 3.1 Screening of potential bolting resistance orphan genes

**Figure 1:**
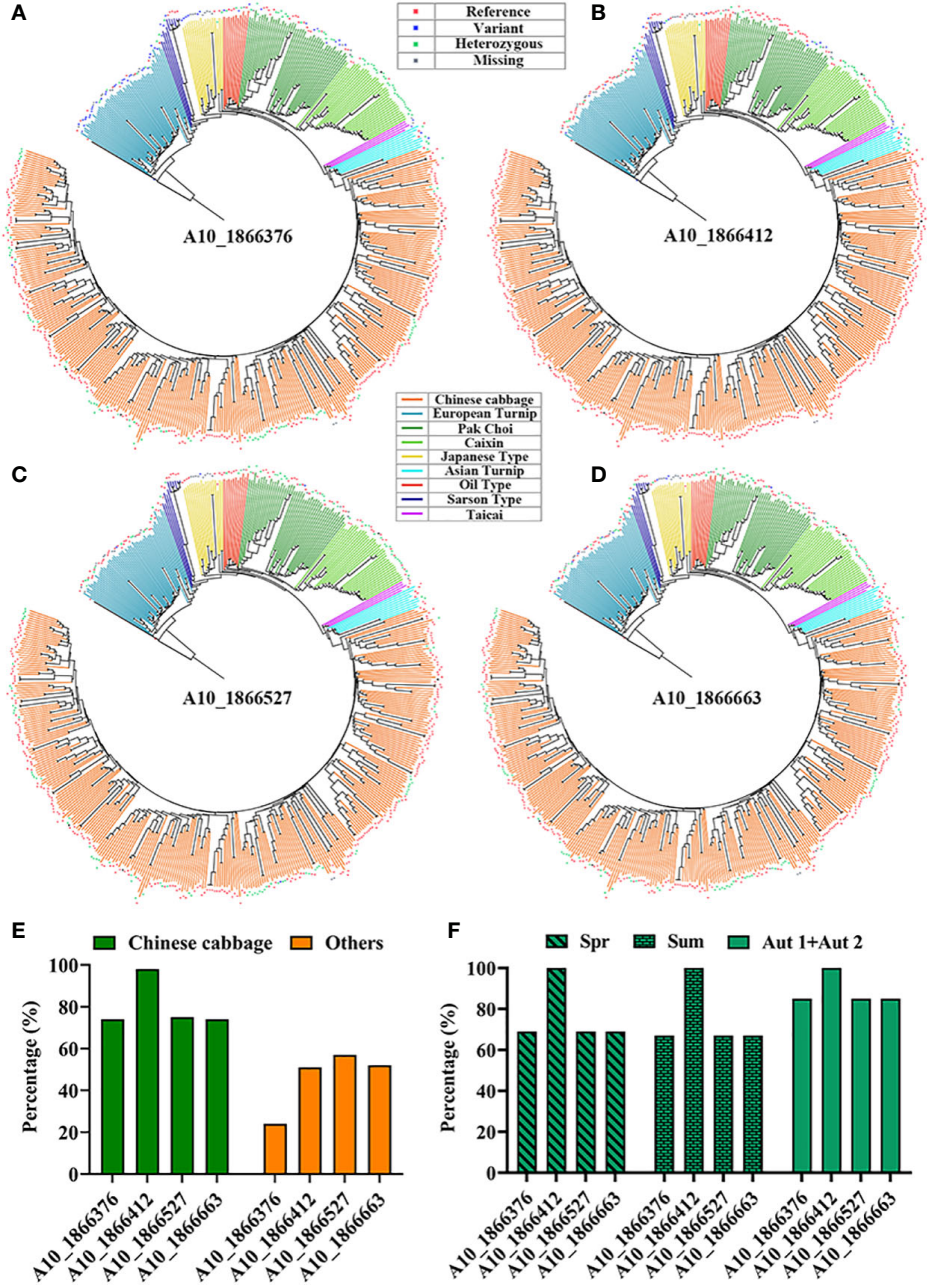
Genotypic analyses of SNPs in the *BR1* gene region. **(A-D)** Different SNP types identified across 524 *B rapa* accessions in the *BR1* gene region. If a particular accession was consistent with the reference genome, this is denoted by a reference designation, whereas the variant designation was used to indicate an accession with a genotype divergent from the reference genome. Deletion events were designated by ‘Missing’, and heterozygous typing was represented by ‘Heterozygous’. **(E)** The percentage of sequence conservation in Chinese cabbage and other accessions. **(F)** Percentage of sequence conservation in different Chinese cabbage ecotypes, with Spr and Sum respectively corresponding to the spring and summer ecotypes, while the autumn ecotypes are denoted by Aut1+Aut2.

Genotypic analyses of structural variations of ten orphan genes were randomly selected from our prior study (Jiang et al., 2018) (**Supplementary Table S2**), which was used to screen potential bolting resistance orphan genes. BRAD was used to perform genotypic analyses of structural variations in this *BR1* gene region across 524 *B. rapa* accessions. There were no any structural variations in four orphan genes (*BraA01g024790.3C*, *BraA02g027570.3C*, *BraA03g056750.3C*, and *BraA09g030600.3C*). The highest average sequence conservation rates was found in the gene region of *BR1* (*BraA10g003580.3C*), including just four single nucleotide polymorphisms (SNPs) in this region (A10_1866376, A10_1866412, A10_1866527, and A10_1866663) (**Figures 1A-D**), which accounting for ~80% in Chinese cabbage relative to ~46% in other accessions (**Figure 1E**). The A10_1866412 SNP exhibited the highest sequence conservation ratio (~98%) in Chinese cabbage, while the average variation ratio in Chinese cabbage was ~20%, with this value being lower than in other accessions (~54%). The A10_1866376 SNP exhibited the highest variation ratio (~76%) in other accessions. Average *BR1* sequence conservation rates were approximately 77%, 75%, and 88% in Chinese cabbage spring, summer, and autumn ecotypes, respectively (**Figure 1F**). No variations in the A10_1866412 SNP were observed among these three Chinese cabbage ecotypes, and the average percentage of variation in these three respective ecotypes was approximately 23%, 25%, and 12%. *BR1* located on chromosome A10 at positions 1866284 – 1866805, and this gene contains no introns and consists of 522 bases that encode a protein 173 amino acids in length. The BR1 protein does not contain any known signal peptides, motifs, or conserved domains, and it was not identified as a TF in subsequent analyses. As such, the *BR1* gene is more conserved in Chinese cabbage than in other accessions, potentially owing to domestication and associated selection for leafy head development and bolting resistance in this economically important species.

### 3.2 *BR1* expression patterns in Chinese cabbage

**Figure 2:**
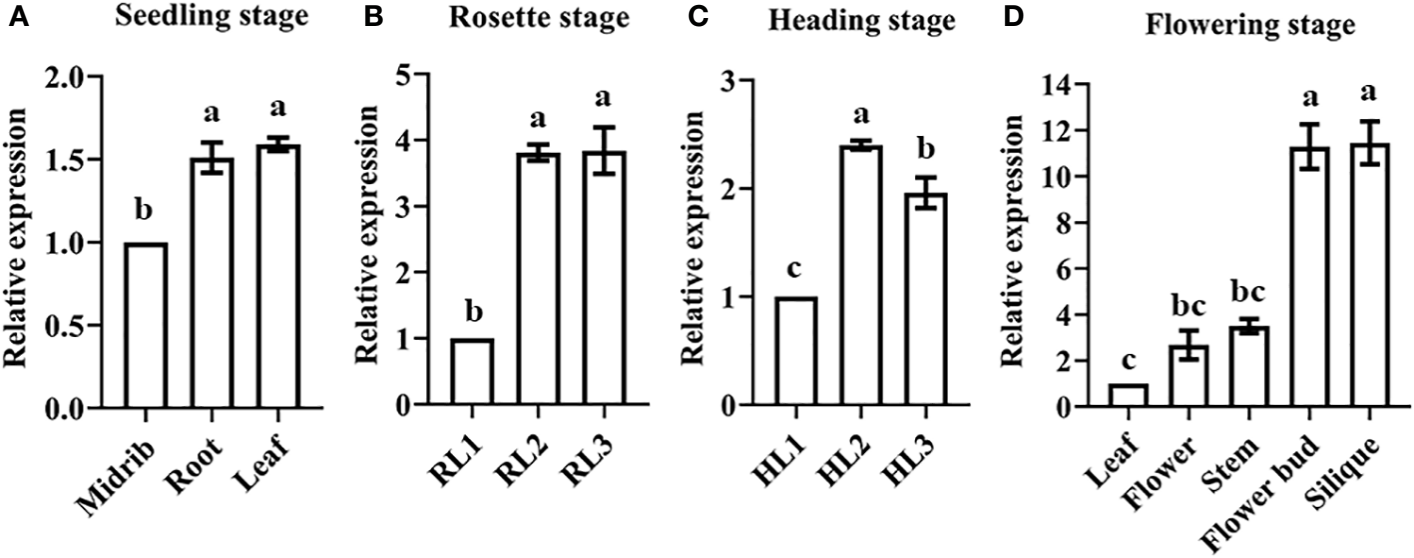
*BR1* expression patterns during different developmental stages in Chinese cabbage. *BR1* expression was assessed in the **(A)** seedling, **(B)** rosette, **(C)** heading, and **(D)** flowering stages. Data are means ± SE of three independent measurements. Statistically distinct groups are marked with black letters (one-way ANOVA, *p* < 0.05).

**Figure 3:**
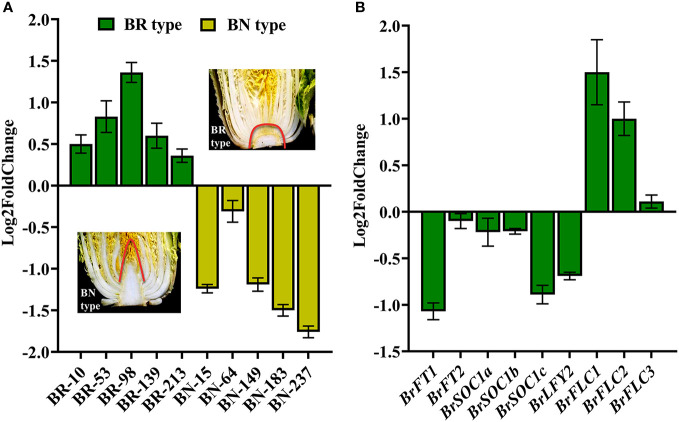
Expression patterns of *BR1* **(A)** and representative mark genes **(B)** in bolting resistant or non-resistant Chinese cabbage during heading stage. Examples of bolting resistant type (BR type) and bolting non-resistant type (BN type) Chinese cabbage were showed in the figure. Green column represented BR type, yellow column indicated BN type. BR type inbred lines ‘BR-98’ was used for expression analyses.

qPCR was used to analyze *BR1* expression patterns in an effort to explore its potential functional roles during different stages of *B. rapa* development. Significant increases in *BR1* expression were observed in both roots and leaves in the seedling stage, with peak expression in the leaves (**Figure 2A**). During the rosette and heading stages, *BR1* expression levels rose significantly in the middle (RL2 and HL2) and inner leaves (RL3 and HL3) relative to the outer leaves (RL1 and HL1) (**Figures 2B, C**). During the flowering stage, higher *BR1* expression levels were detected in the flower buds and silique relative to other analyzed tissues (**Figure 2D**). These results provide a basis for the further exploration of *BR1* as an *OG* associated with the regulation of different stages of vegetative and reproductive growth in Chinese cabbage plants. Next, the expression levels of *BR1* were detected within bolting resistant (BR type) and bolting non-resistant (BN type) Chinese cabbage to explore its functions. Study indicated that Chinese cabbage with the rounded apices of its short stem belonging to BR type, and BN type possessed the pointed apices (Mero and Honma, 1984). Surprisingly, *BR1* possessed increase trends in five lines from BR type, but showed suppressed trends in BN type (**Figure 3A**). Meanwhile, the expression patterns of mark genes were analyzed to confirm the function of controlling bolting resistance in Chinese cabbage. Mark genes including *BrFLCs*, *BrFTs*, *BrSOC1s*, and *BrLFYs*. BR type inbred lines ‘BR-98’ was selected for further analysis. As expected, four *BrFLCs* showed increased trends, while *BrFTs*, *BrSOC1s*, and *BrLFY2* were displayed decreased trends (**Figure 3B**). The expression of *BrFT3* and *BrLFY1* was not detected. These expression patterns correspond to the bolting resistance phenotype of inbred line ‘BR-98’. Such results suggested that *BR1* may directly or indirectly involve in bolting resistance of Chinese cabbage.

### 3.3 *BR1* overexpression regulates bolting resistance in *Arabidopsis*

**Figure 4:**
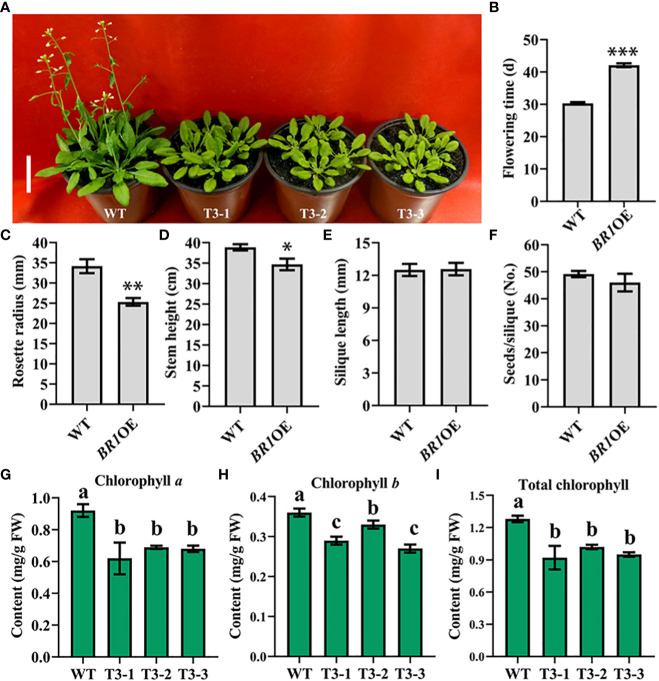
*BR1*OE mutant phenotypic characterization. **(A)** Under LD conditions the *BR1*OE transgenic plant exhibited a late-flowering phenotype. Representative images of 32-day-old transgenic plants from three separate genetic transformation events (T3-1, T3-2, and T3-3) and wild-type Col-0 (WT) are shown. Scale bar: 4 cm. **(B)** Flowering time. **(C)** Rosette radius. **(D)** Stem height. **(E)** Silique length. **(F)** Seed number per silique. Data are means ± SE of three independent measurements. Significantly differences were identified when comparing WT and *BR1*OE plants using Student’s *t*-tests (*p < 0.05, **p < 0.01, ***p < 0.001). **(G)** Chlorophyll *a* content. **(H)** Chlorophyll *b* content. **(I)** Total chlorophyll content. Analyses were performed with transgenic plants from three separate genetic transformation events (T3-1, T3-2, and T3-3) and wild-type Col-0 (WT) *Arabidopsis.* FW: fresh weight. Data are means ± SE of three independent measurements. Statistically distinct groups are marked with black letters (one-way ANOVA, *p* < 0.05).

To better understand the functional roles played by *BR1, A. thaliana* plants overexpressing this gene were prepared. After floral dip transformation, the DsRed marker was used to select T2 homozygous seeds from different self-pollinated T1 transgenic seed lines, and T3 plants were then planted to assess phenotypes following the harvesting of seeds from T2 plants. Phenotypes were compared between the wild-type (WT) and *BR1* overexpressing plants (*BR1*OE) in the vegetative and reproductive phases of growth under LD conditions (16 h of light/8 h of dark). These analyses revealed that *BR1* overexpression strongly delayed *Arabidopsis* floral transition (**Figure 4A**), with *BR1OE* plants exhibiting a 28% delay in flowering time relative to WT controls (**Figure 4B**). Rosette radius was also reduced by ~26% in these *BR1*OE mutant plants (**Figure 4C**), suggesting that *BR1* may regulate leaf elongation under standard growth conditions. *BR1OE* plants also exhibited a decrease in final stem height relative to WT controls (**Figure 4D**), whereas silique length and numbers of seeds per silique were unchanged (**Figures 4E, F**). Notably, *BR1*OE plants exhibited reductions in chlorophyll content relative to WT plants (**Figures 4G-I**). Accordingly, the overexpression of *BR1* enhances *Arabidopsis* bolting resistance.

### 3.4 Analysis of transcriptomic sequencing data and qPCR validation

Transcriptomic sequencing was conducted to explore the molecular mechanisms underlying the effects of *BR1* on delayed flowering time in *A. thaliana* by preparing cDNA libraries from the leaves of WT and *BR1OE* plants. All samples exhibited Q20 values > 97% and Q30 values > 92% (**Supplementary Table S3**). Raw and clean read numbers for individual samples respectively ranged from 43,611,780 - 48,073,534 and 42,257,488 - 46,814,082, consistent with highly reliable transcriptomic detection results.

**Figure 5:**
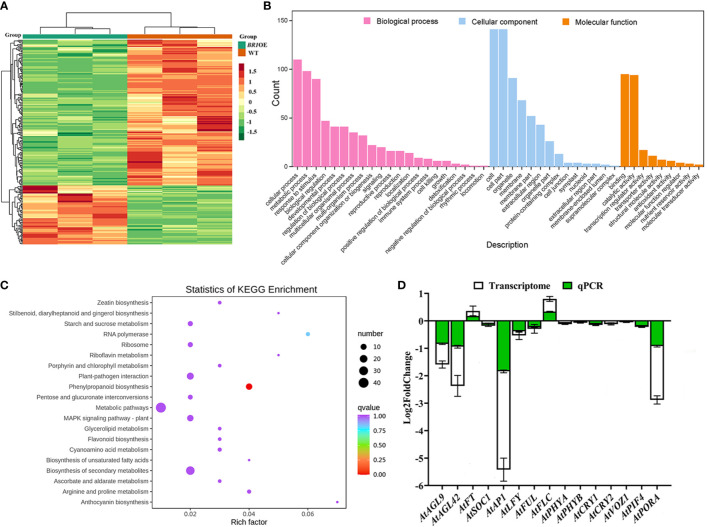
Identification of genes differentially expressed between WT and *BR1*OE plants. **(A)** DEG heatmap analysis. **(B)** DEG Gene Ontology (GO) classification was performed, with individual bars denoting the numbers of DEGs mapped to particular GO categories (pink: biological process; blue: cellular component; orange: molecular function). **(C)** The top 20 most enriched KEGG pathways associated with identified DEGs. Pathways are provided with the corresponding enrichment factors. **(D)** qPCR validation of gene expression profiles.

Relative to WT plants, the *BR1*OE mutants exhibited significantly delayed flowering time. To better understand the transcriptomic changes underlying this phenotype, FPKM values for individual unigenes were compared between these two *A. thaliana* varieties to identify genes that were differentially expressed. In total, 254 DEGs were identified of which 73 and 181 were respectively up- and downregulated (28.74% and 71.26%, respectively) (**Figure 5A**, **Supplementary Table S4**). GO enrichment analyses of these DEGs identified 44 significantly enriched GO terms (**Figure 5B**, **Supplementary Table S5**). These included enriched biological processes (including cellular process, metabolic process, biological regulation, developmental process, regulation of biological process, multicellular organismal progress, signaling, reproductive process, reproduction, growth, and rhythmic process), cellular component (including cell, cell part, organelle, membrane, membrane part, and extracellular region), and molecular function (including binding, catalytic activity, transcription regulator activity, transporter activity, structural molecule activity, antioxidant activity) terms. KEGG pathway enrichment analysis also identified 47 significantly enriched KEGG pathways (**Supplementary Table S6**), with the top 20 for each DEG set being presented in **Figure 5C**. The majority of these DEGs were enriched in pathways including “metabolic pathways and biosynthesis of secondary metabolites (ko01100)”, “plant-pathogen interaction (ko04626)”, “phenylpropanoid biosynthesis (ko00940)”, “plant MAPK signaling pathway (ko04016)”, and “porphyrin and chlorophyll metabolism (ko00860)”.

Next, DEGs associated with the floral transition pathway were compared between WT and *BR1OE* plants, revealing three MADS-box TFs among these DEGs including *AGAMOUS-LIKE 9* (*AtAGL9*, *AT1G24260*), *AtAP1*, and *AGAMOUS-LIKE 42* (*AtAGL42*, *AT5G62165*) (**Figure 5D**). These TFs were significantly downregulated in *BR1OE* plants relative to WT controls, in line with the delayed flowering time phenotype (**Figure 4A**). While not significantly downregulated, other floral integrator genes exhibited downward expression trends in *BR1OE* plants, including *AtSOC1*, *AtLFY*, and *FRUITFULL* (*AtFUL*, *AT5G60910*), whereas *AtFT* was expressed at low levels in both WT and mutant plants. The expression level of floral repressor *AtFLC* gene showed upward trends in *BR1OE* plants as expected. Interestingly, the downward expression trends of six key genes that involved in photoperiodic flowering pathway were observed in *BR1OE* plants, including *PHYTOCHROME A* (*AtPHYA*, *AT1G09570*), *PHYTOCHROME B* (*AtPHYB*, *AT2G18790*), *CRYPTOCHROME 1* (*AtCRY1*, *AT4G08920*), *CRYPTOCHROME 2* (*AtCRY2*, *AT1G04400*), *VASCULAR PLANT ONE ZINC FINGER PROTEIN 1* (*AtVOZ1*, *AT1G28520*), and *PHYTOCHROME INTERACTING FACTOR 4* (*AtPIF4*, *AT2G43010*). Moreover, the key chlorophyll biosynthesis-related gene *Protochlorophyllide oxidoreductase A* (*AtPORA*, *AT5G54190*) was also significantly downregulated in *BR1*OE mutant plants relative to WT controls, explaining the observed reduction in chlorophyll content (**Figures 4G-I**). These results indicated that *BR1* gene may regulate flowering primarily through vernalization and photoperiodic pathway, which further supported a model in which *BR1* serves as an important regulator of *Arabidopsis* floral transition.

### 3.5 BR1 localizes to the nucleus

**Figure 6:**
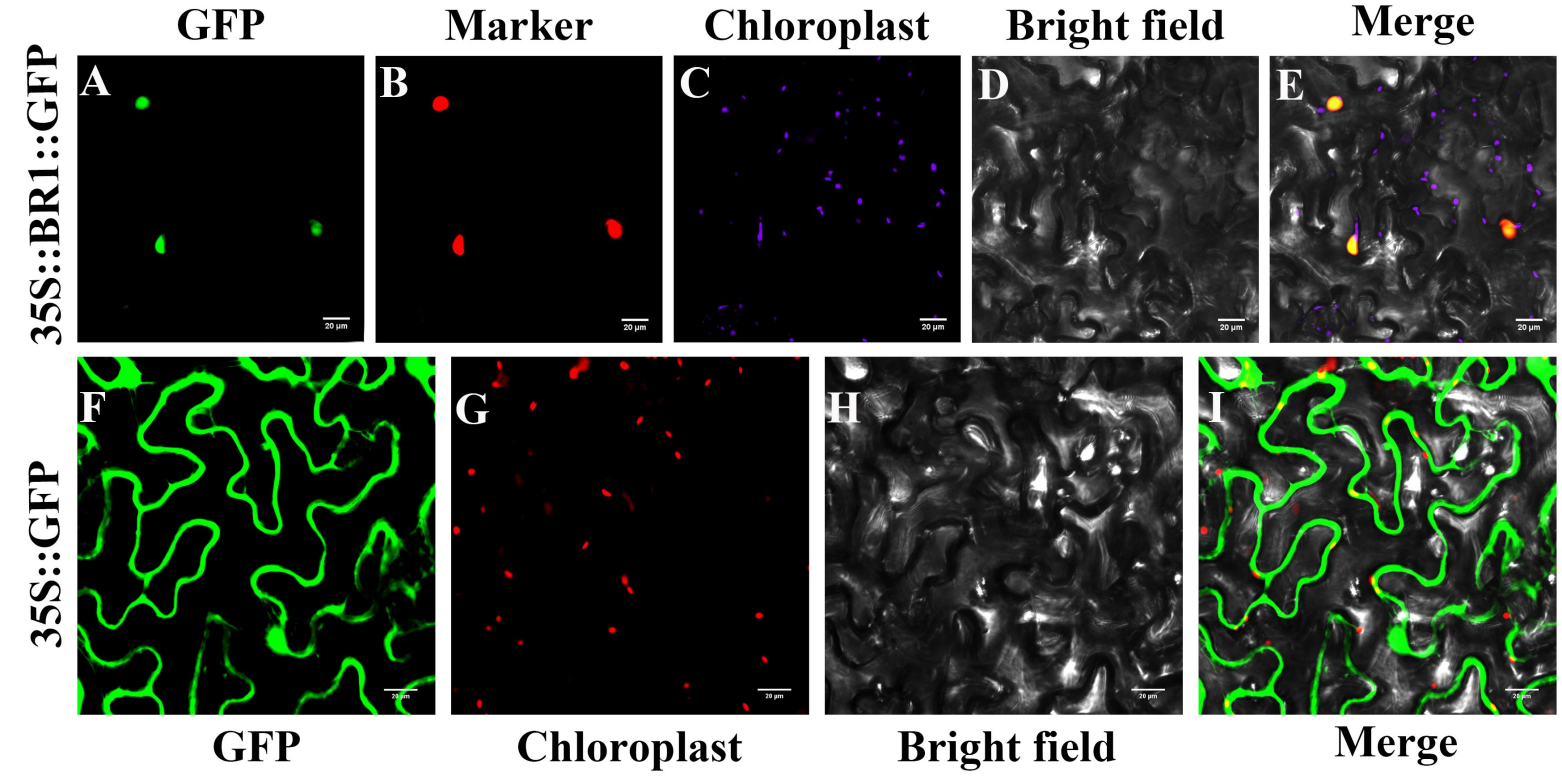
BR1 subcellular localization analyses. **(A, F)** GFP fluorescence. **(B)** Nuclear marker fluorescence. **(C, G)** chloroplast signals. **(D, H)** Brightfield images. **(E, I)** Merged images. A Leica confocal microscope was used to collect images at 48 h following agro-infiltration. Control GFP localization was evident throughout these cells. Scale bar: 20 μm.

A vector encoding the 35S::BR1::GFP fusion protein under the control of the CaMV35S promoter and a control empty 35S::GFP vector were prepared and used to conduct subcellular localization analyses. These assays revealed that BR1-GFP primarily localized to the nucleus, whereas control GFP signal was visible throughout the nuclear and cytosolic compartments (**Figure 6**). BR1-GRP and nuclear marker co-expression revealed complete overlap between these two signals, confirming that BR1 is a protein that localizes to the nucleus.

## 4 Discussion

### 4.1 *BR1* promotes improved bolting resistance

Genotypic analyses of these *OGs* across 524 accessions revealed four SNPs in the *BR1* gene, consistent with the high levels of *BR1* conservation in Chinese cabbage relative to other accessions. This provided preliminary support for the role of this and other *OGs* as regulators of flowering habits, offering insight into the distinct bolting mechanisms that may have evolved in *B. rapa* that are absent in *Arabidopsis* (Jiang et al., 2022). Potential *OG* and lineage-specific gene family contributions to species-specific phenotypes have previously been characterized in the context of multicellular development in dictyostelids (Luna and Chain, 2021). *OGs* are thus associated with species-specific traits, in line with the present results. Despite the close relationships between Chinese cabbage and *A. thaliana*, these plants have evolved certain distinct characteristics over the course of evolution including distinct leaf shape, leaf size, and flowering timing. During the heading stage of growth, Chinese cabbage plants exhibit a leafy head consisting of leaves with extremely inwardly curved blades on their shoot apex, with these leafy heads being composed of many heading leaves that generally curve following the rosette stage (Ren et al., 2018). *BR1* is mainly expressed in leaves, especially in the middle and inner leaves during the vegetative stage, suggesting that *BR1* may play a role in Chinese cabbage leafy head formation. *OGs* also exhibit a high degree of tissue-specific expression in different tissues and stages of development (Donoghue et al., 2011; Jiang et al., 2018), which may reflect the putative functions of *BR1* in the developmental regulation of Chinese cabbage. Well correspondence with delayed flowering phenotype in ‘*BR1*OE’, the expression of *BR1* was increased in BR type, whereas decreased in BN type, which indicating that *BR1* may play a role in bolting resistance of Chinese cabbage. The expression levels of several mark genes were detected in representative bolting resistance inbred line, floral inhibition factor *BrFLCs* displayed upregulated expression, and other flowering integrators showed downregulated expression. Studies indicated that *FLC* homologues in *Brassica* species act similarly to *AtFLC* that can delay flowering (Yuan et al., 2009; Xi et al., 2018). The expression of integration factors was similar to previous study (Dong et al., 2016). These results further verified the bolting resistance phenotype of ‘BR-98’, and reflected the potential role of *BR1* in the regulation of bolting resistance.

### 4.2 Heterogeneous *BR1* expression regulates *Arabidopsis* bolting resistance

Recent years, bolting resistance of Chinese cabbage had attracted more and more attention from scientists. In this study, the *OG BR1* was identified as a novel regulator of plant morphological characteristics. *Arabidopsis BR1*OE mutants exhibited notably delayed flowering time together with significant decreases in stem height, rosette radius, and chlorophyll content. The overexpression of *GRAINS NUMBER 2* (*GN2*) gene unique to the ‘Yuanjiang’ common wild rice genome, was similarly found to promote a later heading date, decreased plant height, and a reduction in grain number relative to WT controls (Chen et al., 2017). *Brassica-Specific Genes 1* (*BSGs1*) has similarly been shown to be specifically expressed in Chinese cabbage during the heading stage, suggesting that it may be related to leafy head formation (Jiang et al., 2018). Flowering time is an agronomic trait that is critically important to Chinese cabbage production, and premature bolting can significantly reduce harvest yields and quality (Yuan et al., 2009). Chinese cabbage bolting and flowering generally necessitate vernalization and photoperiodism (Elers and Wiebe, 1984), and *BR1*-related flowering time delays may be linked to enhanced bolting resistance, highlighting a promising new pathway that can help prevent premature flowering for these economically important Chinese cabbage plants.

### 4.3 *BR1* alters flowering integrator gene expression in *Arabidopsis*

An appropriately timed floral transition is vital to ensuring (Srikanth and Schmid, 2011). By analyzing the transcriptome of the *Arabidopsis BR1*OE overexpression plants, we identified several flowering-related genes responsive to *BR1* overexpression. The transcriptomic sequencing and qPCR analyses performed herein confirmed that *BR1* overexpression in *Arabidopsis* significantly altered the expression of *AtAGL9*, *AtAGL42*, and *AtAP1*, in addition to promoting the downregulation of the floral transition identity genes *AtSOC1*, *AtLFY*, and *AtFUL*. Decreases in the expression of these integrator genes were consistent with observed flowering delays in mutant *BR1*OE plants. AtSOC1 can interact with FRUITFULL (AtFUL) to promote the activation of *AtLFY*, triggering the expression of the floral meristem identity gene *AtAP1* promoting floral commitment such that flowers develop on the SAM (Jaudal et al., 2018). *SPL* TFs can promote flowering time and floral fate, with MADS-box TFs including *AtAP1*, *AtFUL*, and *AtAGL9* serving as regulators of floral fate (Freytes et al., 2021). The *SOC1*-like gene, *AtAGL42*, is linked to the control of SAM and axillary meristem (AM) floral transition (Dorca-Fornell et al., 2011). AtAGL9 was shown to bind to and repress *AtSOC1*, while activating expression of a large number of floral homeotic genes (Srikanth and Schmid, 2011). The expression level of *AtFLC* gene displayed upward trends in *BR1OE* plants as expected. As a major inhibitor of flowering, FLC suppressed the expression of transcription factors (TFs) needed for the SAM floral transition (Cho et al., 2017). Low expression level in *AtFT* was also evident in *BR1*OE plants relative to WT controls, and this phenomenon still needs further clarification. Eight core regulatory genes in photoperiodic flowering pathway showed downward expression trends, including *AtPHYA*, *AtPHYB*, *AtCRY1*, *AtCRY2*, *AtVOZ1*, and *AtPIF4*. These genes regulated flowering, and both of these mutants flower late under different conditions (Simpson and Dean, 2002; Thines et al., 2014). How the overexpression of *BR1* gene affects the expression of these genes still needs further study.

Notably, *BR1* overexpression resulted in significant *AtPORA* downregulation in *Arabidopsis*, consistent with the observed reductions in chlorophyll levels in *BR1*OE transgenic plants (**Figure 4G, H, I**). POR is a key enzyme for the light-induced greening of etiolated angiosperm plants, which is one of three known light-dependent enzymes, catalyses reduction of the photosensitizer and substrate protochlorophyllide to form the pigment chlorophyllide (Reinbothe et al., 2010; Buhr et al., 2017; Zhang et al., 2019). The *Arabidopsis porA-1* mutant plants have been shown to exhibit severe photoautotrophic growth defects and decreases in total chlorophyll content (Paddock et al., 2012), confirming the present results. Through comparisons of transcriptomic profiles, most enriched KEGG pathways were metabolic pathways related to the biosynthesis of secondary metabolites. GO enrichment analyses indicated that all DEGs were related to metabolic, developmental, reproductive, and growth-related processes. Based on the above analysis, we speculated that *BR1* may regulate flowering time delay through vernalization and photoperiodic pathway. However, the mechanisms through which the *BR1* pathway contributes to delayed flowering warrant further research. As BR1 localizes to the nuclear compartment, the identification of upstream regulatory TFs and downstream target proteins can be readily performed through respective yeast one-hybrid and yeast two-hybrid screens. Further knockout and overexpression analyses in Chinese cabbage will thus be essential to fully characterize how *BR1* regulates bolting resistance.

## 5 Conclusions

The highly conserved nature of *BR1* in Chinese cabbage accessions supports its potential role as a regulator of bolting resistance. The expression of *BR1* was increased in bolting resistant Chinese cabbage and decreased in bolting non-resistant type. The overexpression of *BR1* in *Arabidopsis* resulted in delayed flowering time and other obvious phenotypes. Both transcriptomic sequencing and qPCR analyses further revealed that nuclear-located orphan gene *BR1* may function as a new mediator of flowering time through vernalization and photoperiodic pathway. These results offer novel insight into the links between *OGs* and flowering time in *B. rapa*, providing a theoretical basis for future studies aimed at examining the mechanisms governing bolting resistance in this economically important species.

## Data availability statement

The datasets presented in this study can be found in online repositories. The names of the repository/repositories and accession number(s) can be found below: PRJNA922732 (SRA).

## Author contributions

MJ and HL conceived and designed the experiments. MJ, YZ, and HL performed the experiments. MJ, YZ, XY, XL, and HL analyzed the data. MJ and HL drafted the manuscript. All authors contributed to the article and approved the submitted version.
